# Genetic basis of mycotoxin susceptibility differences between budding yeast isolates

**DOI:** 10.1038/s41598-017-09471-z

**Published:** 2017-08-23

**Authors:** Xtopher Quispe, Sebastián M. Tapia, Carlos Villarroel, Christian Oporto, Valentina Abarca, Verónica García, Claudio Martínez, Francisco A. Cubillos

**Affiliations:** 1 Centro de Estudios en Ciencia y Tecnología de Alimentos (CECTA), Universidad de Santiago de Chile (USACH), Santiago, Chile; 2 Millennium Nucleus for Fungal Integrative and Synthetic Biology (MN-FISB), Departamento de Genética Molecular y Microbiología, Facultad de Ciencias Biológicas, Pontificia Universidad Católica de Chile, Casilla, 114-D Santiago, Chile; 3 Departamento de Ciencia y Tecnología de los Alimentos, Universidad de Santiago de Chile (USACH), Santiago, Chile; 4 Departamento de Biología, Facultad de Química y Biología, Universidad de Santiago de Chile, Santiago, Chile

## Abstract

Micophenolic acid (MPA) is an immunosuppressant mycotoxin which impairs yeast cell growth to variable degrees depending on the genetic background. Such variation could have emerged from several phenomena, including MPA gene resistance mutations and variations in copy number and localisation of resistance genes. To test this, we evaluated MPA susceptibility in four *S. cerevisiae* isolates and genetically dissected variation through the identification of Quantitative Trait Loci. Via linkage analysis we identified six QTLs, majority of which were located within subtelomeres and co-localised with *IMD2*, an inosine monophosphate dehydrogenase previously identified underlying MPA drug resistance in yeast cells. From chromosome end disruption and bioinformatics analysis, it was found that the subtelomere localisation of *IMD2* within chromosome ends is variable depending on the strain, demonstrating the influence of *IMD2* on the natural variation in yeast MPA susceptibility. Furthermore, GxE gene expression analysis of strains exhibiting opposite phenotypes indicated that ribosome biogenesis, RNA transport, and purine biosynthesis were impaired in strains most susceptible to MPA toxicity. Our results demonstrate that natural variation can be exploited to better understand the molecular mechanisms underlying mycotoxin susceptibility in eukaryote cells and demonstrate the role of subtelomeric regions in mediating interactions with the environment.

## Introduction

Fungal infections are a major concern in cereal crops, tree nuts, and vegetable production, partly due to the vast number of toxic metabolites that can contaminate plants and food products. These fungi cause several plant, animal, and human diseases affecting not only production yields but also human health. Indeed, it has been estimated that over 25% of agricultural commodities in the world are contaminated with fungal infections^1, 2^. Becoming a general threat for human health, toxic fungal secondary metabolites, also known as mycotoxins, can be transmitted along the food chain where they can cause carcinogenic and mutagenic diseases in humans. The main foodborne mycotoxins of public health concern are aflatoxins, fumonisins, ochrartoxins, patulin, tricothecenes, deoxynivalenol, micophenolic acid and zearalenone, and these are primarily produced by fungi of the genera *Aspergillus*, *Fusarium* and *Penicillium* ^3^. The yeast *Saccharomyces cerevisiae* has been widely used for hundreds of years as a starter culture in the beverage industry^4^. Many studies have reported the presence of mycotoxins during the fermentation of beer and wines^5^; this, suggests a long-lasting interaction between yeast and mycotoxins, and *S. cerevisiae* is likely highly tolerant to these toxins.

Mycophenolic acid (MPA) is a mycotoxin commonly produced in silage by *Penicillium roqueforti* and has antifungal^6^, antibacterial and antiviral activities^7^. This toxin is frequently used as an immunosuppressant due to its ability to inhibit inosine 5′-monophosphate dehydrogenase (IMPDH), an enzyme directly involved in guanine nucleotide biosynthesis^8^. T and B lymphocyte proliferation is inhibited in the presence of this toxin due to MPA’s ability to block *de novo* purine biosynthesis^9^. Mammals, like humans and mouse, have two copies of IMPDH and are susceptible to MPA. In contrast, the model organism *S. cerevisiae* contains four copies of this enzyme and is resistant to high concentrations of this toxin^10^. Given this, genetic studies in yeast have taken advantage of its ability to naturally resist MPA in order to extensively describe the IMPDH enzymes. *S. cerevisiae* IMDH genes are named from *IMD1* to *IMD4*, possess high sequence similarity and have been shown to perform distinct functions within the purine biosynthesis pathway^11^. *IMD2* gene expression is induced in the presence of MPA, and *IMD2* knock-outs are MPA susceptible^12^. The role of the paralogous genes *IMD3* and *IMD4* in MPA resistance is less well documented, and mutants have not been reported to be affected by this toxin. Similarly, *IMD1* is a non-functional protein and has not been associated with MPA resistance. In the S288c strain, *IMD1* and *IMD2* genes are localised within subtelomeres, while *IMD3* and *IMD4* are core genes.

The majority of large high-throughput studies of MPA resistance (and many other toxins) in yeast have been performed using the heterothallic strain S288c^10^. Thus, most of the information currently available in databases has been derived from this unique genetic pool. However, this and many other related groups of strains do not represent the extensive diversity found in budding yeast^13–17^. In this context and with the aim of increasing the information available for more strains, several re-sequencing projects have obtained full genome sequences for hundreds of strains^18–23^. Moreover, high-throughput phenotyping efforts have provided highly resolved maps of natural trait variation in yeast, and it has been found that phenotypic diversity is well correlated with genetic diversity^24^. Most yeast traits, including resistance to antibiotics, chemicals, carbon source use, and fungicide resistance are genetically complex, determined by multiple quantitative trait loci (QTL)^13, 15, 25, 26^. The majority of the QTLs described thus far mapped within core regions of the genome, however a fraction of these QTLs have been reported to map within subtelomeric regions^13, 17, 27^. For example, advances inter-cross lines between two *S*. *cerevisiae* industrial strains with divergent performance at low temperature showed the presence of four genomic regions involved in the adaptation at low temperature, three of them located in the subtelomeric regions^27^. In contrast, other phenotypes such as nitrogen consumption during wine fermentation have mapped all QTLs to core regions within the genome^28^, demonstrating that subtelomeric localisation of QTLs is dependent on the environmental condition evaluated.

In part, this structural variation between strains is due to gene copy number variation and localisation in different subtelomeres, providing the species with a greater capacity to respond to environmental fluctuations^29^. For example, copy number variation and the subtelomeric localisation of the *ARR* gene cluster has been implicated in differences in arsenite resistance across naturally occurring isolates^13^. In this context, IMDH subtelomeric localisation could impact MPA resistance across strains and could confer differences in resistance within the species.

Changes in gene expression are among the major forces affecting adaptation and evolution^30^. Differences in the abundance of transcripts are common between individuals and are due to: *cis* (local) and/or *trans-*factors (over long genetic distances). These two types of factors impact gene expression differently. Variants explaining a greater fraction of expression differences between alleles are usually placed near the encoded transcript. *Cis*-regulatory factors impact transcription in several ways, such as by affecting regulatory elements, including transcription factor (TF) binding sites and chromatin regulators that impact transcription by altering chromatin structure at promoter regions^31–36^. In contrast, genetically distant *trans-*variants tend to explain less expression variance, but affect a greater number of target genes^16, 37, 38^.

Here, we sought to identify genetic variants that confer resistance to MPA in naturally occurring *S. cerevisiae* isolates and to determine differences in gene expression among these isolates when exposed to this mycotoxin. Linkage analysis demonstrated the presence of a series of QTLs, the majority of which were located within subtelomeres and correlated to the presence of functional *IMD2* alleles spread across different chromosome ends, endorsing the potential role on environmental adaptation of the extensive gene-reshuffling found in subtelomeres among yeast strains. Moreover, insights into the physiological response of different genotypes to micophenolic acid susceptibility are provided via extensive gene expression profiling, including the identification of GxE interactions.

## Results

### Differences in MPA resistance in naturally occurring *S. cerevisiae* isolates

**Figure 1:**
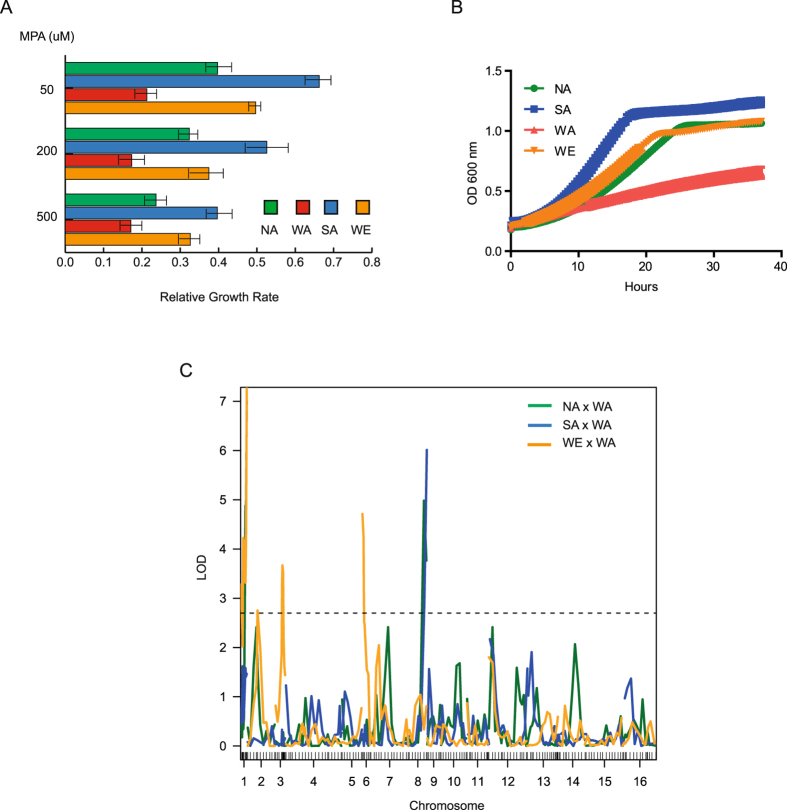
Growth rates of *S. cerevisiae* isolates subjected to MPA. (**A**) Relative growth rates (MPA/YPD) for the North American (NA), West African (WA), Sake (SA), and Wine/European (WE) isolates. (**B**) Growth data for the microcultivation of four isolates grown in 200 μM MPA. (**C**) LOD plot for linkage analysis for the NA × WA (green), SA × WA (blue) and WE × WA (orange) crosses. Dotted line represents the highest LOD cut-off scores (LOD = 2.73) among the three crosses. FDR = 0.05 was used to determine significance.

In order to identify patterns of phenotypic diversity for MPA resistance in naturally occurring *S. cerevisiae* isolates, we measured quantitative growth when isolates were exposed to an MPA treatment in microscale liquid cultivation. For this, representative isolates of previously described clusters from North America (YPS128, NA), Sake (Y12, SA), Wine/European (DBVPG6765, WE) and West Africa (DBVPG6044, WA) were used^18, 19^. Strains were grown in media containing 50 μM to 500 μM MPA, and growth rates were estimated (Table S1). Overall, MPA treatment decreased growth rates by more than 50% in all isolates, clearly demonstrating the detrimental effect of this mycotoxin on yeast fitness (Fig. 1A). However, we observed a wide range of differences among the isolates depending on the MPA concentration administered (low concentration – 50 μM or high concentration - 500 μM) (Fig. 1A); overall, the SA strain was the most resistant, while the WA strain was susceptible even to low MPA concentrations. Similarly, the NA and WE strains had relatively high growth rates regardless of the MPA concentration used, indicating that both types of isolates are highly resistant (Fig. 1B).

The WA strain, which was the most susceptible type of isolate in our study, was previously used for the generation of F1 recombinant populations. Thus, to investigate the genetic architecture of MPA resistance in *S. cerevisiae* we used the following crosses based on a star-cross design and performed linkage mapping: WA × NA, WA × SA, and WA × WE^13^. For this, 96 segregants from each recombinant population were grown in micro-cultures containing 200 μM MPA, and growth rates were obtained for each individual segregant (Table S2). Subsequently, we performed linkage analysis using previously published genotype information^13^ and found six QTLs distributed in chromosomes I, III, VI and VIII (Fig. 1C, Table S3). Based on overlapping intervals, these QTLs were summarised into four major QTL regions (QTL1 – QTL4) that explained from 16.1% to 30% of the total phenotypic variance depending on the cross (Table S3). For all QTLs, the WA variant was detrimental to MPA resistance, as seen based on the phenotypic pattern of the WA parental strain. Interestingly, QTL1, QTL3, and QTL4 were located in subtelomeric regions, where QTL1 and QTL4 co-localise with *IMD1* and *IMD2*, respectively, in the reference genome.

### *IMD2* localisation across different subtelomeres validates MPA resistance differences between strains

**Figure 2:**
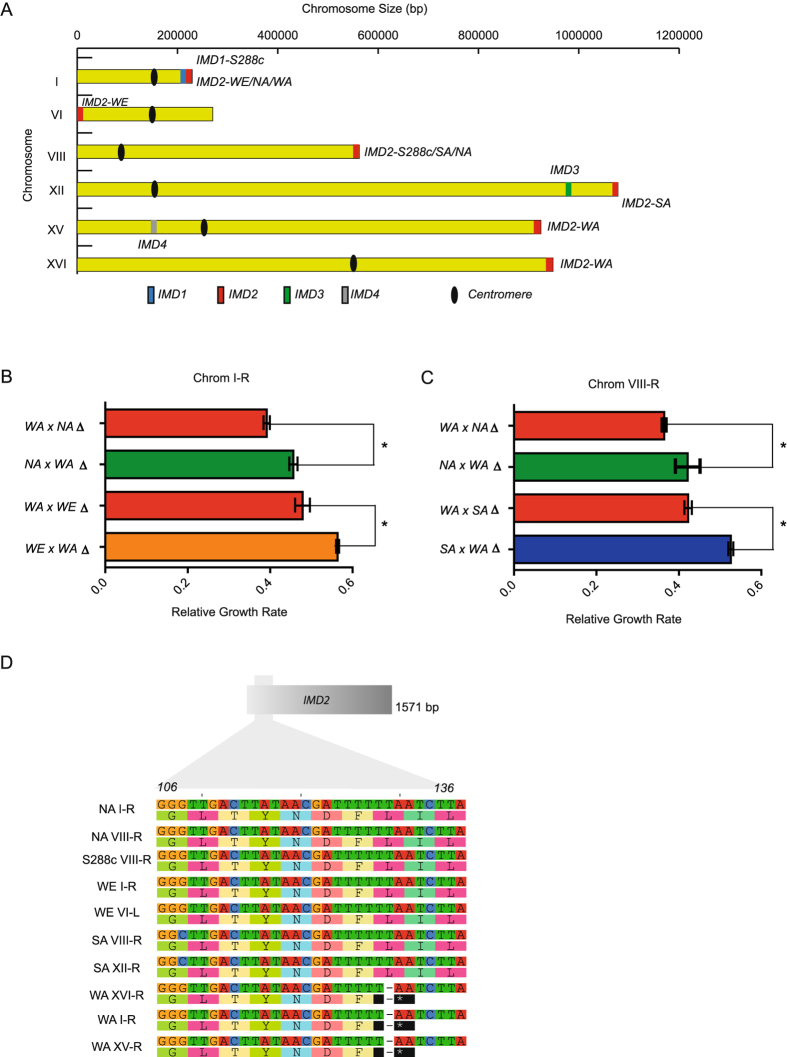
Mapping of the *IMD* family. (**A**) Physical localisation of *IMD1* (blue rectangle), *IMD2* (red rectangle), *IMD3* (green rectangle), and *IMD4* (grey rectangle) across the genomes of the four isolates. Abbreviations denote each isolate type and the black circle depicts the centromere localisation. (**B**) Relative growth rates (estimated as growth rate of isolate subjected to MPA/growth rate of isolate in YPD) of reciprocal hemizygotes for subtelomeres I-R and (**C**) VIII-R (*) denotes a significant difference according to a Student’s t-test p-value < 0.05. (**D**) Nucleotide and amino acid Imd2p sequence in the WE, WA, SA, NA and S288c reference isolates, (*) denotes a stop codon.

To identify and validate the role of *IMD* genes underlying MPA resistance differences between strains, we used publically available yeast genome sequence data^29^ and manually searched and curated *IMD* copies across the four strains genomes and compared our subtelomeric QTLs with the chromosome ends that could contain any of the *IMD* genes in the four isolate types. Our bioinformatics analysis allowed us to find several rearrangements between strains, specifically, *IMD2* co-localised with QTL1,QTL3 and QTL4 (Fig. 2A). More specifically, QTL1 co-localised with *IMD2* in NA, WA and WE at subtelomere I-R (Chromosome I- right); with QTL3 in WE at subtelomere VI-L; and with QTL4 in SA and NA at subtelomere VIII-R. Interestingly, *IMD2* was not previously annotated in I-R (WA), VI-L (WE), VIII-R (NA) and XV-R (WA), suggesting the presence of different *IMD2* copies within these subtelomere and demonstrating the power of the linkage analysis approach to decipher new subtelomeric gene-reshuffling. QTL2 did not co-localised with any of the *IMD* genes, suggesting an alternative molecular mechanism, in this case, underlying MPA resistance.

Subsequently, we disrupted the I-R (Chromosome I – right) in WA, NA and WE strains and VIII-R subtelomeres in WA, NA and SA isolate types and generated reciprocal hemizygotes between the WA strain and the rest of the corresponding strains for the ends of both chromosomes (Fig. 2B and C). In agreement with the subtelomere assemblies^29^, we observed significant differences in the relative growth rates (estimated as MPA/YPD) of the WA × NA and WA × WE hemizygotes when subtelomere I-R was disrupted, where hemizygotes carrying the WA copy were susceptible to MPA (Table S4, Fig. 2B). From these results it can be seen that the presence of *IMD2* within the I-R subtelomere in the WE and NA strains provides MPA resistance, while WA’s *IMD2* I-R would be non-functional. Indeed, we found three *IMD2* copies in the WA isolate localised in I-R, XV-R and XVI-R, where all of them contained a premature stop codon at position 42, hence encoding for a potential non-functional protein (Fig. 2D). Similarly, when the same assay was performed using the VIII-R disruption, the WA × SA and WA × NA hemizygotes exhibited different growth rates when subjected to MPA, with hemizygotes carrying NA and SA *IMD2* alleles showing greater growth rates under MPA compared to those containing the WA variant (Fig. 2C); this demonstrates the role of functional *IMD2* alleles within the VIII-R subtelomere in the SA and NA isolates.

### Gene expression differences between West African (WA) and Sake (SA) isolates demonstrate greater ribosome biogenesis in the SA isolate

**Figure 3:**
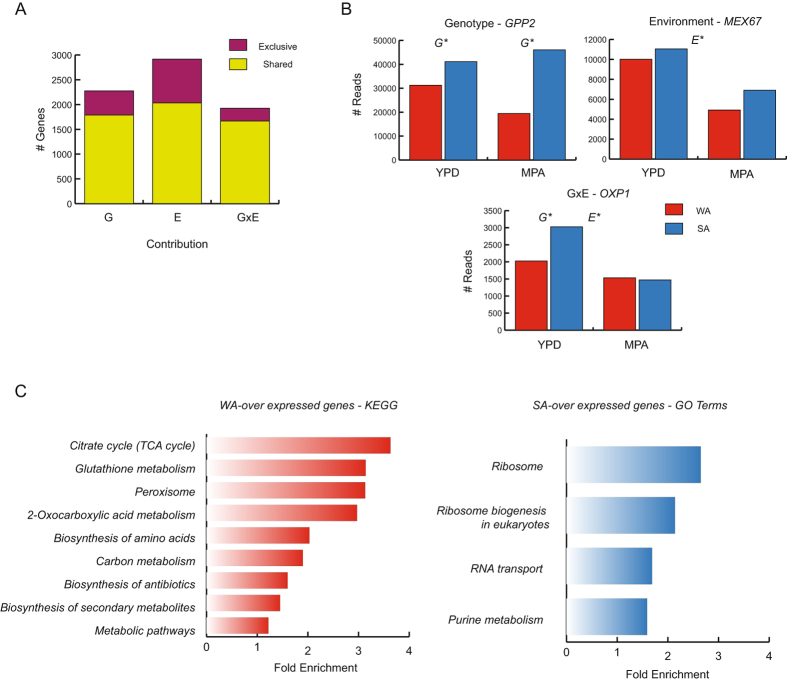
GxE interactions in WA and SA parental strains grown in MPA. (**A**) Number of genes exhibiting significant Genotype (G), Environment (E) and GxE (Genotype × Environment) components derived from parental expression data. The number of genes with shared (genes with more than one significant component, in yellow) and exclusive (exhibiting a sole significant component, in purple) components are depicted. (**B**) Examples of WA and SA reads in YPD and MPA for *GPP2* (Genotype), *MEX67* (Environment) and *OXP1*(GxE) examples, (G*) and (E*) denote significant genotype and/or environment effects FDR < 0.05. (**C**) KEGG Pathway enrichment of WA over-expressed genes (left red panel) and SA over-expressed genes (right blue panel).

Changes in gene expression are important contributors to phenotypic differences. After investigating the localisation of genes that confer major resistance to MPA, we sought to further understand the mechanisms by which WA and SA strains differ and respond to this mycotoxin to untangle regulatory divergence between strains. For this, initially we estimated gene expression levels via RNA-seq. This was done for both strains grown under two conditions: i) Control treatment (Rich YPD media) and ii) MPA treatment (200 μM). For each sample, we obtained RNA during the exponential growth phase (O.D. ~ 0.8); mRNA was sequenced to obtain an average of nearly 18 million reads, which were subsequently aligned against the S288c reference genome (see methods). Next, we assessed genome-wide divergence for 4,761 genes which were expressed in all genotypes and treatments (Table S5a). Then, we applied a GxE analysis (using edgeR, see methods) in order to identify genes differentially expressed due to genotype differences (G), due to environmental differences (i.e. treatment) independent of genotype (E), and those due to the interaction of both variables (GxE). Overall, we found 2,276; 2,916; and 1,925 differentially expressed genes due to G, E and GxE factors, respectively; this demonstrates the relevant impact of MPA treatment on gene expression (Fig. 3A). Moreover, out of this, G, E or GxE factors exclusively influenced gene expression in 489, 884, and 258 genes, respectively. For example, *GPP2* which encodes a DL-glycerol-3-phosphate phosphatase involved in glycerol biosynthesis was highly expressed in SA in comparison to WA, independent of the treatment (Fig. 3B). On the contrary, *MEX67*, which encodes an RNA binding protein involved in nuclear mRNA export, was highly expressed in isolates subject to control treatment compared to those subjected to MPA, independent of the genotype of the strain (log_2_ YPD/MPA = 0.34, Fig. 3B). On the other hand, *OXP1*, a gene encoding a 5-oxoprolinase enzyme highly expressed during DNA replication as a response to stress, was found over-expressed in SA and to a lesser extent in WA; this pattern, however, was only found when isolates were grown in the control media (log_2_ = −0.61 WA/SA, FDR = 0.04) and not when they were grown in the presence of MPA (log_2_ = 0.21 WA/SA, FDR = 0.2, Fig. 3B), suggesting a strong down-regulation of *OXP1* in SA, but not in WA in response to MPA.

In order to better determine which metabolic pathways are enriched by these subsets of genes and to obtain further biological insights for on-going processes within each isolate type, we looked for Gene Ontology (GO) term enrichment within the GxE genes. For this, the set of 1,925 genes that were significant according to the GxE analysis were categorised as either WA > SA (959 genes) or SA > WA (964 genes) over-expressed transcripts due to MPA treatment. We found that over-expressed WA genes were enriched for GO terms related with primary defence against oxidative damage, such as peroxisome biogenesis and glutathione metabolism (Fig. 3C, Table S5b). Moreover, vacuolar transport has been indicated as an alternative MPA resistance mechanism. Indeed, *TPO1*, a gene encoding for a polyamine transporter of the major facilitator superfamily, was overexpressed in WA > SA (Table S5), suggesting a compensatory mechanism for the lack of *IMD2*. On the contrary, SA over-expressed genes were highly enriched for genes involved in ribosome biogenesis, RNA transport, and purine biosynthesis (Table S5c); it is thus seen that the SA GTP deficit could be counterbalanced by augmenting ribosome biogenesis. Altogether, these results suggest that MPA massively impacts the sensitive isolate type (WA) in terms of guanine biosynthesis, protein translation, and ribosome translocation.

We next sought to identify significantly enriched transcription factor binding motifs within regulatory regions of genes that showed a response to MPA treatment. For this, we scanned the set of genes for which there were significant GxE effects, and in particular, those genes that were over-expressed in the WA strain (959 genes). For this, we used the expert-curated set of transcription factor (TF) motifs from the YeTFaSCo database^39^. Overall, we found 45 transcription factor binding sites that were highly represented among these regions (*P*-value < 0.002 - Bonferroni correction, Table S6), and among these, Msn2p, a stress-responsive transcriptional activator, was the top-score, suggesting that Msn2p could be the main transcription factor responding to MPA treatment in the WA isolate.

### Robust cis-response of individuals in control and MPA treatments

**Figure 4:**
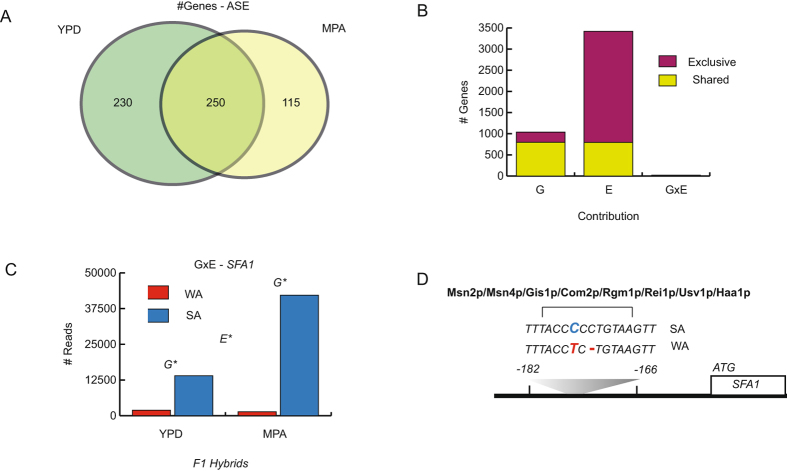
ASE and GxE interactions in F1 Hybrids. (**A**) Number of Genes exhibiting ASE in individuals subjected to YPD (green) and MPA (yellow) treatments. (**B**) Number of genes exhibiting significant Genotype (G), Environment (E) and GxE (Genotype × Environment) components derived from F1 hybrid expression data. The number of genes with shared (genes with more than one significant component, in yellow) and exclusive (exhibiting a sole significant component, in purple) components are depicted. (**C**) Number of WA and SA reads of F1 hybrids grown in YPD and MPA; *SFA1* is given as an example of a gene affected by both genotype and environment (GxE), (G*) and (E*) denote significant genotype and/or environment effects FDR < 0.05. (**D**) The sequence divergence between the SA and WA strains is between −182 and −166 bp from the *SFA1* ORF. Also, the predicted binding differences due to the two polymorphisms are depicted.

Differences in expression levels between genotypes and between individuals subjected to different environmental treatments could be due to *cis* or *trans*-factors and could provide insights into the molecular changes within regulatory regions that underlie environmental adaptation. In order to evaluate genome-wide differences in the *cis*-response upon MPA treatment, we estimated Allele Specific Expression (ASE) using RNA-seq in the WA × SA hybrid. RNA-seq data was compared across treatments and we evaluated the allele specific expression of 4,761 genes expressed in the hybrid and parental strains (Table S7a). A total of 480 and 365 genes were found to exhibit significant *cis*-regulatory differences (FDR < 0.05) under control (YPD) and MPA treatments, respectively; and out of these, 250 genes were shared across treatments (Fig. 4A), suggesting a robust *cis*-response regardless of treatment.

To further investigate the *cis*-response of individuals subjected to different environments and to estimate the proportion of *cis* genes responding to environmental stimuli, we performed a GxE analysis using edgeR and the hybrid data. We accounted for the effects of genotype (G), environment (E) and genotype × environment (GxE) (Fig. 4B, Table S7b). Overall, we observed 1,039 alleles that were differently expressed depending on genotype (G), independent of their response to environment. Additionally, 3,420 alleles were differently expressed depending on the environmental treatment (E) that the hybrid was subjected to. Contrarily, we only found a low number of genes (20) for which reads counts differed between both alleles and across conditions due to the interaction between genotype and environmental effects (GxE; FDR < 0.05). For example we observed in *SFA1*, a gene encoding for a bi-functional alcohol dehydrogenase and formaldehyde dehydrogenase that increases abundance of proteins related with DNA replication stress, a greater number of SA reads in response to MPA in the hybrid compared to control treatment (Fig. 4C), likely due to polymorphisms within the regulatory region specifically responding to MPA treatment. Indeed, six polymorphisms were found between both strains in the *SFA1* regulatory region (200 bp immediately upstream of ATG) where a single deletion in WA (del −172C) could impact transcription factor binding of stress response regulators, such as Mns2p and Msn4p (Fig. 4C). However, *SFA1* represents a particular case, and rather the majority of genes displayed a specific response to environmental stimuli, independent of the genotype.

## Discussion

Micophenolic acid is a potent mycotoxin that inhibits inosine 5’-monophosphate dehydrogenase (IMPDH), which is involved in guanine nucleotide biosynthesis^40^. This mycotoxin has been commercially used as an inmunosuppresor and MPA activity has shown clinical efficacy for patients with autoimmunity^41^. In this context, the presence of this toxin in silage and other food products represents an important threat for human and animal health, directly affecting the individual’s immune system. Smaller eukaryotes are similarly affected by MPA’s ability to impair cell growth; thus, *S. cerevisiae* has served as a prevailing model to understand the mechanisms of MPA action^10^. Yeast possesses four *IMD* genes, where *IMD2* has been the single ORF implicated in MPA tolerance.

In our assay, we found extensive growth differences between four natural *S. cerevisiae* isolates exposed to MPA. Specifically, the WA isolate was highly sensitive compared to other isolate types, suggesting significant genetic differences between strains underlying MPA resistance (Fig. 1). To untangle genetic variants underlying MPA resistance differences among these strains, we used a star design recombinant population where the strain most sensitive to MPA (WA) was used as the central parent and crosses with the three other strains were used. This proven approach has been successfully applied to map the genetic variation responsible for quantitative traits in Arabidopsis^42, 43^. This design maximises genetic variation across contributing strains and eases the phenotyping of the entire set of segregants. Indeed, in our study four QTLs were found in the recombinant populations, and three of these were co-localised with *IMD2*, located within the subtelomeric regions of chromosome I, VI and VIII. Subtelomeres harbour a significant percentage of the natural variation that exists between strains, and have been found to contain many genes that mediate interactions with the environment. As such, up to 25% of QTLs in yeasts have been mapped to these regions^13, 17, 27^. Moreover, these genomic areas are highly variable compared to core chromosomal regions as the presence and absence of genes together with the number of copies and localisation within subtelomeres varies greatly between strains^17, 29^. A recent subtelomere quantitative comparison between yeast isolates has demonstrated the dynamic nature of these regions in *S. cerevisiae* strains; specifically, these regions are characterised as having accelerated evolution, rapid gene reshuffling, and greater dn/dS rates compared to central regions. Indeed, our linkage and bioinformatics sequence analysis approaches identified several QTLs co-localising with *IMD2* copies across chromosome ends in the different genetic backgrounds (Fig. 2A). These results agree with those of Yue *et al*. who have suggested the presence of *IMD2* genes in chromosome I-R of WE and NA strains. Similarly, we found an *IMD2* copy in the VIII-R subtelomere of SA, in agreement with Yue’s data. However, the VI-L *IMD2* copy in the WE strain, the I-R and XV-R in WA together with the VIII-R in NA were not previously reported and were identified in this work (Fig. 2A). Interestingly, WA possess three *IMD2* copies in different subtelomeres, all of them exhibiting a premature stop codon (Fig. 2D). In this context, subtelomeric deletions on chromosome I and VIII of the different strains analysed here demonstrated the presence of functional *IMD2* copies in the NA and WE and SA and NA isolates, respectively, mediating MPA resistance, while it also shows a disrupted *IMD2* copy in WA potentially encoding for a non-functional protein and explaining MPA susceptibility (Fig. 2B). All these results demonstrate the dynamic nature of the different *IMD2* copies and the extensive gene re-shuffling of these alleles across subtelomeres, conferring variable MPA resistance levels between strains.

Differences in gene regulation play an important role in speciation and adaptation. Recently, the advent of new sequencing technologies has allowed for the characterisation and study of gene expression and associated regulatory mechanisms in a large number of individuals and species at unprecedented scales^44^. In this context, natural variation in gene expression represents a key factor shaping an individual’s phenotype, where polymorphisms within e.g. transcription factor binding sites can lead to phenotypic differences between strains^33–36, 45^. Moreover, in recent years the regulation of gene expression has been at the forefront of genetic and evolutionary studies in multiple species. To complement our linkage analysis, we implemented allele-specific expression profiling of WA and SA phenotypically divergent isolates in order to estimate the contribution of *cis*-regulatory variants to gene expression variability. Moreover, we have performed a GxE analysis to determine MPA action mechanisms and how gene expression and interactions differ depending on the genotype. Genome-wide estimates of ASE and transcript abundance were obtained using RNA-seq read counts from individual F1 hybrids and parents exposed to high MPA concentrations. ASE estimates of isolates subjected to the control and MPA treatment were robust, indicating a dominant *cis*-effect independent of the environment and predominantly dependent on the genotype (Fig. 3). Studies in higher eukaryotes, such as those involving human cell lines and Arabidopsis, have shown that environment specific *cis*-regulatory effects are rare^46, 47^, in agreement with our assay of different environmental conditions. A similar study in yeast employing a rich media treatment and an ethanol treatment has shown that *cis*-variants are generally more stable across environments, as opposed to *trans*-variants which are more likely to vary depending on environmental conditions^38^. Nevertheless, we found extensive expression differences between parental strains, likely due to distal-factors, allowing us to gain insight into MPA action mechanisms. Previous studies have reported greater *IMD2* expression levels in yeast cells exposed to MPA^11, 48^. These antecedents agree with our observations of greater *IMD2* transcript levels in WA and SA strains in the presence of MPA, however the premature stop codon within this gene in WA hinders a robust response against this mycotoxin in this isolate type. An alternative MPA resistance mechanism is the over expression of *TPO1* ^48^, a gene encoding for a polyamine transporter of the major facilitator superfamily and located in the vacuolar membrane^49^. Indeed, our study demonstrates that WA over-expresses *TPO1* respect to the SA isolate, suggesting a higher number of vacuolar transporters and a greater MPA accumulation in the vacuole in the WA strain.

Interestingly, in our study the majority of under-expressed GxE genes in the sensitive isolate type were enriched in GO terms related to ribosome biogenesis, RNA transport, and purine metabolism, suggesting a deficit in these functions. MPA impacts GTP – GDP and GMP biosynthesis and thus, a series of cellular processes, including the elongation phase of transcription, which is dependent on GTP^50^. Subsequently, mutants in genes involved in transcription elongation, such as: *DST1*, *RTF1*, *RPB9* and *PAF1* are MPA susceptible^48^. We observed that all of these genes are overexpressed in the SA isolate in the presence of MPA, indicating that most compensatory mechanisms are active and proving MPA resistance in this strain. Moreover, this isolate type has greater expression levels in genes related with ribosome biogenesis, likely due as a response to the GTP deficit, augmenting ribosome levels and protein synthesis^11, 48^. Altogether, these mechanisms contribute to withstand the long-term MPA exposure in the SA strain.

Our linkage analysis and RNA-seq assay results provide, to our knowledge, the broadest evidence of the genetics and biology underlying differences in MPA resistance among yeast individuals. The use of a finely resolved subtelomere sequence for these strains, together with our own bioinformatics search, allowed us to pinpoint *IMD2* gene localisation variation across distal chromosomal regions and provide biological evidence of how this variation impacts MPA resistance. Moreover, studying regulatory variation using RNA-seq has helped resolve MPA action mechanisms; specifically, we have determined that MPA could impact ribosome biogenesis & RNA transport by causing GTP deficits. Defining the genetic architecture and molecular mechanisms by which gene re-shuffling and polymorphisms within coding and regulatory regions impact gene expression represents a critical stride to understanding the origin of phenotypic differences between individuals.

## Materials and Methods

### Yeast strains and media culture

Haploid parental strains Y12 (named as Sake, ‘SA’, Mat alpha ho::HygMX, ura3::KanMX), YPS128 (named as North American, ‘NA’, Mat alpha ho::HygMX, ura3::KanMX), DBVPG6044 (named as West African, ‘WA’, Mat alpha ho::HygMX, ura3::KanMX) and DBVPG6765 (named as Wine/European, ‘WE’, Mat a, ho::HygMX, ura3::KanMX), together with the WA × SA hybrid and 288 segregants (96 per F1 cross: WA × NA, WA × SA & WA × WE) used in this study (for the WE × SA, WE × NA and WE × WA crosses) have been described previously^13, 51^.

### Growth assays and QTL mapping

The microcultivation phenotyping assay was performed as previously described^25^. Briefly, parental strains and segregants were precultivated in 200 μL of YNB medium supplemented with uracil (0.67% yeast nitrogen base, 2% glucose, 0.2% uracil) for 48 h at 28 °C. For the experimental run, segregants were inoculated to an optical density (OD) of 0.03–0.1 (wavelenght 630 nm) in 200 uL of media and incubated without agitation at 28 °C for 24 h (YNB control) and 36 h (MPA treatment) in a Tecan Sunrise absorbance microplate reader. OD was measured every 20 minutes using a 630 nm filter. Each experiment was performed in duplicate. Growth rates for each strain and trait were calculated as previously described^52^. Briefly, OD measurements versus time were fitted to the re-parameterized Gompertz equation proposed by Zwietering *et al*.^53^ from which growth rates were obtained. Finally, relative growth rates were estimated as MPA growth rate/YPD growth rate.

QTL mapping was performed as previously described^25^. Briefly, LOD scores were estimated using a non-parametric model and the significance of each region was determined from permutations. Briefly, for each trait and cross, we permuted the phenotype values 1000 times, recording the maximum LOD score each time. We called a QTL significant if its LOD score was greater than the 0.05 tail of the 1000 permuted LOD scores. The percentage of phenotypic variance explained for a QTL was calculated using the following formula, where n represents the sample size:

$${\rm{Percentage}}\,{\rm{of}}\,{\rm{variance}}\,{\rm{explained}}=100(1\mbox{--}{10}^{(-2{\rm{LOD}}/{\rm{n}})}).$$

### Subtelomere sequence analysis and disruption

Bioinformatic analysis to identify *IMD2* sequences were performed using Geneious 8.1.5. and the four genome sequences previously released^29^. Reciprocal hemizygotes were generated from each corresponding parental strain. In all cases the *URA3* gene was used as a selectable marker, and subtelomeres were disrupted as previously described^13^. For this, transformations were performed as described in Jara, *et al*.^54^ where haploid versions of the parental strains were used to delete chromosome ends and construct all the possible combinations of single deletions in the hybrid. For this, mutated parental strains were crossed to generate the reciprocal hemizygote strains and were selected for on double drug plates (50 mg/mL Hygromycin B and 100 mg/mL Nourseothricin). Diploid hybrid strains were confirmed using *MAT* locus PCR^55^, and the subtelomeric deletions were confirmed by PCR using the primer pairs A1/S8. A1 primers are listed in Table S8, while S8 has been previously described elsewhere^56^. Phenotypic differences between strains were compared using a classical Student’s t-test (p-value < 0.05).

### RNA Sequencing

WA and SA haploid parental strains and the WA × SA hybrid were grown in triplicate in 5 mL of rich YPD and YPD supplemented with 200 μM MPA media at 28 °C up to the mid-log phase (OD600, ~ 0.8). Cultures were harvested by centrifugation and cells were treated with 2U of Zymolyase for 30 min at 37 °C. RNA was extracted using the E.Z.N.A. Total RNA Kit I (OMEGA) according to the supplier’s instructions. RNA samples were then treated with DNase I (Promega) to remove genomic DNA traces and total RNA was recovered using the GeneJET RNA Cleanup and Concentration Micro Kit (Thermo Scientific). RNA integrity was confirmed using a Fragment Analyzer. Sequencing was performed in a Illumina HiSeq. 4000. Gene transcript data can be obtained from the Biosample Database Project # PRJNA386475.

RNA-sequencing and bioinformatics were carried out as previously described^25^ with modifications. Briefly, sequences were first assessed for their quality using the FASTQC tool kit (http://www.bioinformatics.babraham.ac.uk/projects/fastqc/). Low quality reads were discarded using the Trimmomatic tool using default score settings and a phred score cut-off of 30 (http://www.usadellab.org/cms/?page=trimmomatic). Parental RNA-seq reads were then aligned to the S288c reference genome (*S. cerevisiae* genome obtained on 03/03/2016, from the Saccharomyces Genome Database, FTP SITE: http://downloads.yeastgenome.org/sequence/S288C_reference/genome_releases/ corresponding to a stable release from January 2015) using Tophat with default score settings^57^. BAM files were sorted and indexed using Samtools with default score settings^58^. Alignments were then processed and gene counts were attained using HTSeq.^59^ with the no-stranded and -gene count configurations from the S288c gff file. Differential expression and GxE was assessed with edgeR using the nbinomTest function and the glmFit function, respectively^60^.

Hybrid RNA-seq reads were analysed as previously described^36^. Briefly, RSEM^61^ was used to align reads to reference transcriptomes, containing the union of the parental transcriptomes and using Bowtie as aligner tool^62^. Reads were subsequently filtered for those that prefered one parental allele over the other (i.e. the read contained one or more bases that differentiated the alleles). Allele specific reads were then summed for each allele and used in subsequent analyses. Alleles with less than 10 reads for all replicates were excluded from all subsequent analyses to avoid mapping biases. Overall, for the gene expression analysis (of parents and hybrids), we excluded 1,846 genes due to the absence of polymorphisms, missing ORFs, low gene/allele counts, or undefined bases in the genome.

### KEGG Pathway enrichment and Transcription factor binding enrichment

Using the DAVID Bioinformatics Resource^63^, differentially expressed genes (either in the parental or ASE assays) were used to test for significant enrichment of genes in pathways listed in the Kyoto Encyclopedia of Genes and Genomes (KEEG). FDR was estimated using the default option^64^. We selected significantly over-represented categories using a FDR < 0.05%.

Transcription factor binding enrichment was performed using the YeTFaSCo database^39^, where the probability of each promoter being bound by each TF (motif occurrence – expert curated option) in each strain was calculated. A rank-sum test was performed and p-value cut-off was chosen based on Bonferroni correction.

### Accession code

The RNA-seq reads from this study are available from NCBI under BioProject ID PRJNA386475.

## Electronic supplementary material

Supplementary Information

Supplementary Dataset 1

Supplementary Dataset 2

Supplementary Dataset 3

Supplementary Dataset 4

Supplementary Dataset 5

Supplementary Dataset 6

Supplementary Dataset 7

Supplementary Dataset 8
