# Supplementary material for: Genetic basis of mycotoxin susceptibility differences between budding yeast isolates
Source: Sci Rep. 2017 Aug 23;7:9173. doi: 10.1038/s41598-017-09471-z (PMC5569051; doi:10.1038/s41598-017-09471-z)
Supplement: Supplementary file 1 — Supplementary Information [file 41598_2017_9471_MOESM1_ESM.pdf]

SUPPLEMENTARY INFORMATION

Genetic basis of mycotoxin susceptibility differences between budding yeast isolates

Xtopher Quispe[1,2], Sebastián M. Tapia[1,2], Carlos Villarroel[1,2], Christian Oporto[1,2], Valentina Abarca[1], Verónica García[1,3], Claudio Martínez[1,3] & Francisco A. Cubillos[1,2,4]

[1]Centro de Estudios en Ciencia y Tecnología de Alimentos (CECTA), Universidad de Santiago de Chile (USACH), Santiago, Chile.

[2]Millennium Nucleus for Fungal Integrative and Synthetic Biology (MN-FISB), Departamento de Genética Molecular y Microbiología, Facultad de Ciencias Biológicas, Pontificia Universidad Católica de Chile, Casilla 114-D, Santiago, Chile.

[3] Departamento de Ciencia y Tecnología de los Alimentos, Universidad de Santiago de Chile (USACH), Santiago, Chile.

[4]Departamento de Biología, Facultad de Química y Biología, Universidad de Santiago de Chile, Santiago, Chile.

Corresponding author: francisco.cubillos.r@usach.cl

Table S1. Relative growth rates of parental strains subjected to the MPA treatment.

Table S2. Growth rates of segregants subjected to either the control (YPD) or MPA treatment (200 uM MPA).

Table S3. QTLs obtained from the linkage map.

Table S4. Relative growth rates of reciprocal hemizygotes' subjected to MPA. The second genotype on each row denotes the deleted allele.

Table S5. Contribution of GxE effects on WA and SA parental strains grown in YPD and MPA. (A) Genotype (G), Environment (E) and Genotype x Environment (GxE) effects on the gene expression of WA and SA parental strains grown in YPD and MPA. Log2 values are shown as WA/SA. (B). KEEG pathways enriched in parental strain genes overexpressed (WA>SA) due to GxE effects. (C) KEEG pathways enriched in parental strain genes overexpressed (SA>WA) due to GxE effects.

Table S6. Transcription factor motif enrichment of WA parental strain genes' affected by GxE.

Table S7. ASE in F1 Hybrids. (A) ASE values (B) GxE of F1 Hybrids.

Table S8. Primer list used in this study.
